# Cohort profile: A Prospective Household cohort study of Influenza, Respiratory syncytial virus and other respiratory pathogens community burden and Transmission dynamics in South Africa, 2016–2018

**DOI:** 10.1111/irv.12881

**Published:** 2021-07-23

**Authors:** Cheryl Cohen, Meredith L. McMorrow, Neil A. Martinson, Kathleen Kahn, Florette K. Treurnicht, Jocelyn Moyes, Thulisa Mkhencele, Orienka Hellferscee, Limakatso Lebina, Matebejane Moroe, Katlego Motlhaoleng, Francesc Xavier Gómez‐Olivé, Ryan Wagner, Stephen Tollman, Floidy Wafawanaka, Sizzy Ngobeni, Jackie Kleynhans, Azwifari Mathunjwa, Amelia Buys, Lorens Maake, Nicole Wolter, Maimuna Carrim, Stuart Piketh, Brigitte Language, Angela Mathee, Anne von Gottberg, Stefano Tempia, for the PHIRST group

**Affiliations:** ^1^ Centre for Respiratory Diseases and Meningitis National Institute for Communicable Diseases of the National Health Laboratory Service Johannesburg South Africa; ^2^ School of Public Health, Faculty of Health Sciences University of the Witwatersrand Johannesburg South Africa; ^3^ Influenza Division Centers for Disease Control and Prevention Atlanta Georgia USA; ^4^ Influenza Program Centers for Disease Control and Prevention Pretoria South Africa; ^5^ United States Public Health Service Rockville Maryland USA; ^6^ Perinatal HIV Research Unit Medical Research Council (MRC) Soweto Matlosana Collaborating Centre for HIV/AIDS and Tuberculosis Tygerberg South Africa; ^7^ Center for Tuberculosis Research, Division of Infectious Diseases, School of Medicine Johns Hopkins University Baltimore Maryland USA; ^8^ Department of Science and Technology/National Research Foundation Centre of Excellence for Biomedical Tuberculosis Research University of the Witwatersrand Johannesburg South Africa; ^9^ MRC/Wits Rural Public Health and Health Transitions Research Unit (Agincourt), School of Public Health, Faculty of Health Sciences University of the Witwatersrand Johannesburg South Africa; ^10^ Division of Medical Virology National Health Laboratory Service, Charlotte Maxeke Johannesburg Academic Hospital Johannesburg South Africa; ^11^ School of Pathology, Faculty of Health Sciences University of the Witwatersrand Johannesburg South Africa; ^12^ Climatology Research Group, Unit for Environmental Science and Management, School of Geo and Spatial Science North‐West University Potchefstroom South Africa; ^13^ Environment and Health Research Unit South African Medical Research Council Johannesburg South Africa; ^14^ Environmental Health Department, Faculty of Health Sciences University of Johannesburg Johannesburg South Africa; ^15^ MassGenics Duluth Georgia USA

**Keywords:** burden, cohort profile, influenza, respiratory syncytial virus, South Africa, transmission

## Abstract

**Purpose:**

The PHIRST study (Prospective Household cohort study of Influenza, Respiratory Syncytial virus, and other respiratory pathogens community burden and Transmission dynamics in South Africa) aimed to estimate the community burden of influenza and respiratory syncytial virus (RSV) including the incidence of infection, symptomatic fraction, and to assess household transmission.

**Participants:**

We enrolled 1684 individuals in 327 randomly selected households in a rural and an urban site over three consecutive influenza and two RSV seasons. A new cohort of households was enrolled each year. Participants were sampled with nasopharyngeal swabs twice‐weekly during the RSV and influenza seasons of the year of enrolment. Serology samples were collected at enrolment and before and after the influenza season annually.

**Findings to Date:**

There were 122 113 potential individual follow‐up visits over the 3 years, and participants were interviewed for 105 783 (87%) of these. Out of 105 683 nasopharyngeal swabs, 1258 (1%) and 1026 (1%) tested positive on polymerase chain reaction (PCR) for influenza viruses and RSV, respectively. Over one third of individuals had PCR‐confirmed influenza each year. Overall, there was influenza transmission to 10% of household contacts of an index case.

**Future Plans:**

Future planned analyses include analysis of influenza serology results and RSV burden and transmission. Households enrolled in the PHIRST study during 2016–2018 were eligible for inclusion in a study of SARS‐CoV‐2 transmission initiated in July 2020. This study uses similar testing frequency to assess the community burden of SARS‐CoV‐2 infection and the role of asymptomatic infection in virus transmission.

**Strengths and limitations of this study**

- PHIRST was conducted in urban and rural African settings with high HIV prevalence, allowing assessment of the effect of HIV on community burden and transmission dynamics of respiratory pathogens.
- Households were selected randomly to provide a representative sample of the community. Twice‐weekly sampling from each cohort of individuals for 6–10 months irrespective of symptoms allows estimation of community burden, household secondary infection risk, and serial interval including asymptomatic or paucisymptomatic episodes.
- Polymerase chain reaction testing of >100 000 nasopharyngeal swab samples for multiple pathogens (influenza, respiratory syncytial virus, pertussis and *Streptoccocus pneumonia*) allows detailed examination of disease burden and transmission and pathogen interactions
- PHIRST was not powered to assess severe outcomes (i.e. hospitalisation and death).
- We only examined four pathogens, but other micro‐organisms may be important. Samples have been stored which could allow us to implement broader multi‐pathogen testing in the future.

## 1 INTRODUCTION

In 2015, lower respiratory tract infections caused an estimated 2.7 million deaths globally.^1^ Among children aged <5 years, the highest mortality rates are in sub‐Saharan Africa where the HIV‐epidemic has increased morbidity of severe pneumonia. Influenza, respiratory syncytial virus (RSV), pertussis and pneumococcus are among the leading causes of pneumonia globally.^2^, ^3^, ^4^, ^5^

Approximately 30% of influenza and RSV transmission is estimated to occur within households.^6^, ^7^ Data on community burden and transmission of respiratory pathogens are important to guide vaccination strategies such as reduced pneumococcal conjugate vaccine dose schedules,^8^ optimal timing of booster doses^9^ and vaccinating community transmitters.^10^, ^11^ Illness episodes in the community may be associated with substantial community impact including absenteeism from school or work and loss of income.^12^

The PHIRST study aimed to estimate the community burden of influenza and RSV (including the incidence of infection and symptomatic fraction) and to assess household transmission of influenza and RSV (Table S1). Secondary objectives included describing the community burden and transmission of *Streptococcus pneumoniae* and *Bordetella pertussis*, estimating the impact of HIV infection and age on disease burden, estimating rates of tuberculosis infection and transmission and investigating the interaction between respiratory viruses and bacteria. We also aimed to evaluate the role of asymptomatic influenza and RSV infection in household transmission.

## 2 COHORT DESCRIPTION

### 2.1 Study population and household eligibility criteria

**FIGURE 1:**
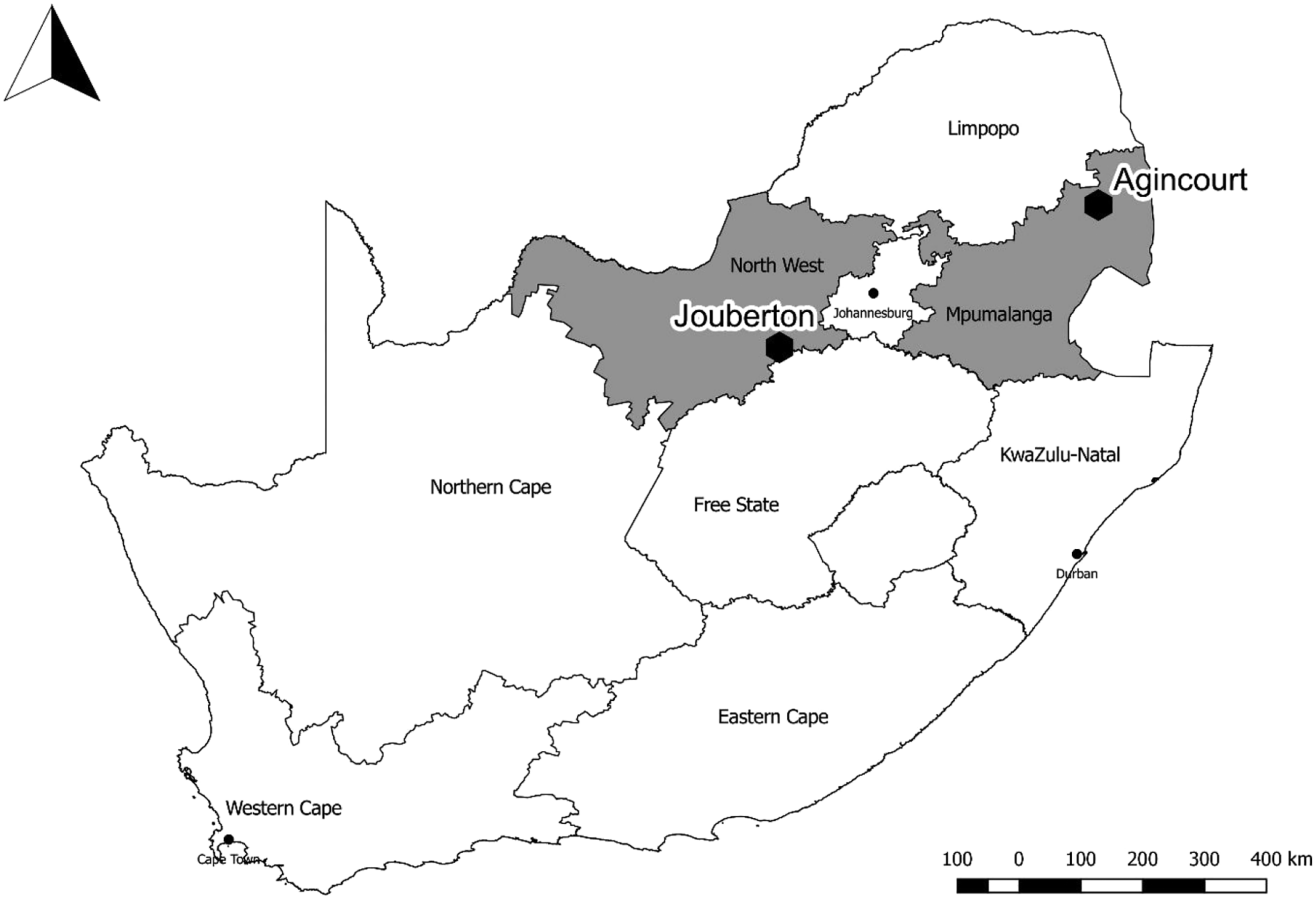
Location of rural (Agincourt) and urban (Jouberton) study sites in South Africa

A prospective cohort study of randomly selected households in South Africa was conducted in a rural and an urban site, each with established surveillance for pneumonia and influenza‐like illness (Figure 1).^13^, ^14^ The rural site in Mpumalanga Province (Agincourt subdistrict) is part of a health and socio‐demographic surveillance system (HDSS), including approximately 116 000 people in 31 contiguous villages.^15^, ^16^ Approximately 30% of the population are former Mozambicans who migrated there in the 1980s.^15^ The urban site, Jouberton Township in Klerksdorp, is part of the municipality of Matlosana in North West Province, with a population of approximately 180 000 people. Mining of gold and uranium, although declining, remains a primary driver of the local economy.^17^

We aimed to enroll approximately 1500 individuals (approximately 500 individuals per year) over three consecutive influenza and RSV seasons to allow the estimation of 20% risk of infection and a 10% risk of illness with 95% confidence intervals (CIs) and 5% absolute precision. Assuming an average household size of five individuals and a loss to follow‐up of 10%, based in previous studies,^18^ we aimed to enroll approximately 55 households with >2 household members per site each year with at least 50% having at least one child aged <5 years in the house.

In rural Agincourt, each year, we purposively selected two different villages within the HDSS. Within these villages, we randomly selected households with >2 members from an enumerated list obtained from the HDSS. In urban Jouberton township, we generated a list of 450 random global positioning system (GPS) coordinates located within a polygon defining the township boundaries using Google Earth. Study staff navigated to the location represented by the coordinates and selected the nearest house. If there was no dwelling within 30 m, the coordinates were discarded. Households were approached consecutively until the desired sample size was reached. If a household withdrew during January–April of each year, it was replaced by a new household, selected consecutively, for the remaining follow‐up period.

At each household with >2 members, study staff requested permission from the head of household to inform members about the study purpose, risks and benefits. If the head of household was a minor or unavailable after three attempts, the household was excluded. Written informed consent was required to participate in the study from all household members aged ≥18 years; assent was required from children aged 7–17 years, and consent from a parent/guardian of children aged <18 years. We included households where ≥80% of household members consented.

### 2.2 Frequency of follow‐up

**FIGURE 2:**
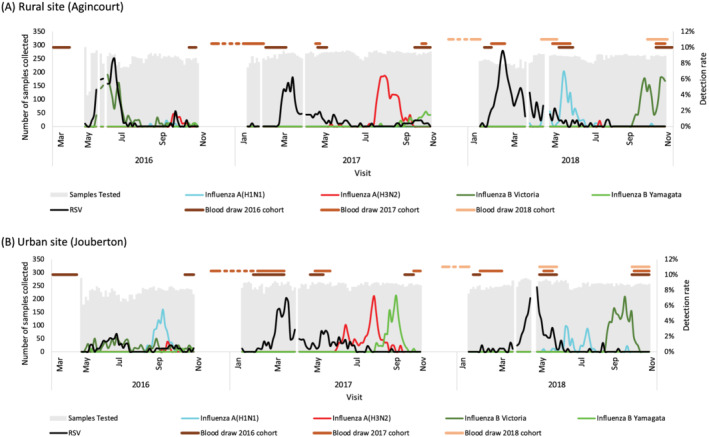
Weekly numbers of nasopharyngeal samples tested and influenza and respiratory syncytial virus (RSV) detections and timing of serology blood draws, a rural and an urban site, South Africa, 2016–2018

Each year, a new cohort of individuals was enrolled and following enrolment, all participants and households had a period of active twice‐weekly follow‐up for 6–10 months. During the year of active follow‐up, participants received twice‐weekly scheduled follow‐up visits (once during Monday–Wednesday and again during Thursday–Saturday) to the household during May–October in 2016 (due to delayed start in the first study year), and January–October in 2017 and 2018 (Figure 2) for the collection of symptom data and nasopharyngeal swabs.

Household surveys were conducted once during the follow‐up period to evaluate household income, housing quality, oropharyngeal carriage of meningococcus, *Corynebacterium diphtheriae* and Group A streptococcus and presence of *S. pneumoniae* DNA in blood by polymerase chain reaction (PCR). Serum samples were also collected at enrolment, before the influenza season, and at the end of the active follow‐up period. In addition, sera were also collected from the 2016 and 2017 cohorts in subsequent years (Figures 2 and S1). Environmental assessments including respirable particulate matter and temperature were undertaken twice a year (summer and winter) (Table 1).

**TABLE 1:** Data and specimens collected on exposures and outcomes in the PHIRST study, South Africa, 2016–2018

Type of data	Details	Frequency
Data collection tool	Data collected	
Enrolment form	Household characteristics (number of individuals and rooms, water source, electricity, smoking in house, cooking in house and relationships)	Enrolment
Case intake form	Age, sex, education, occupation, daily contacts, smoking, alcohol, hand washing, past medical history, documented vaccinations, HIV status and treatment	Enrolment
Household income form	Total household income category	Annual survey
Housing quality checklist	Type and condition of dwelling, roofing, ceiling, walls, windows, floors, temperature control, security, type of toilet, dampness, cooking and space heating fuels, ventilation and pets	Annual survey
Environmental assessment	Respirable particulate matter (PM_4_), carbon dioxide, temperature and relative humidity)	Bi‐annual survey (aim for 1 in winter and 1 in summer)
TB form	Cough, night sweats or weight loss for >2 weeks	Monthly TB visits
Follow‐up visit form	Symptoms (cough, fever, sore throat, runny nose, headache, body pains, difficulty breathing, chest pain, vomiting and diarrhoea), medication, outpatient visits, pharmacist, traditional healer, hospitalisation and death	Twice‐weekly follow‐up visits
Proximity survey	Number and duration of contact events (≤1.5 m apart) between participants wearing sensors	Proximity surveys (4 in 2018)
Contact diary	Age, gender and household member of each individual participant had contact with as well as type of contact, where contact took place and duration of contact	One day in August – October period 2018
Time use survey	Activity and location for each hour of the day	One day in August – October period 2018
Costing survey	Out of pocket costs related to illness among individuals reporting symptoms	October–November 2018
Specimens	Tests performed	
Nasopharyngeal flocked swabs	PCR for influenza, RSV, *B. pertussis*, and *S. pneumoniae*	Enrolment and twice‐weekly follow‐up visits
Clotted blood	Serology for influenza, RSV, pertussis HIV testing for consenting patients	Enrolment and at blood draws (2–3 times per year)
EDTA blood	CD4+ T cell count and HIV viral load for HIV‐infected individuals	Enrolment and end of year
Blood drop	Rapid HIV test for consenting patients	Enrolment
Urine	Quantitative cotinine	Enrolment
EDTA blood	Pneumococcal *lytA* PCR	Annual survey
Oropharyngeal flocked swabs	*C. diptheriae, N. meningitidis* and *Streptococcus pyogenes*	Annual survey
Expectorated sputum	*M. tuberculosis* culture, *B. pertussis* PCR	Monthly TB visits if symptoms of tuberculosis

Abbreviations: EDTA, ethylenediamine tetraacetic acid; PCR, polymerase chain reaction; RSV, respiratory syncytial virus; TB, tuberculosis.

### 2.3 Baseline, symptom and health contact data

Data were collected using REDCap (Research Electronic Data Capture)^19^. Following enrolment, a baseline questionnaire was completed for each household including information on household members, relationships, sleeping arrangements and housing. For each individual, we collected baseline information on demographics, underlying illnesses, vaccinations and occupation. During the twice‐weekly follow‐up phase, at each visit, for each participant, a questionnaire assessing presence of symptoms, absenteeism and health system contacts was completed and nasopharyngeal (NP) swabs were collected regardless of the presence or absence of symptoms (Table 1). Field workers were trained in the identification of respiratory signs and symptoms at the beginning of each year. Symptoms assessed at twice‐weekly visits included fever (self‐reported or measured tympanic temperature ≥38°C), cough, difficulty breathing, sore throat, nasal congestion, chest pain, muscle aches, headache, vomiting or diarrhoea. In 2017 and 2018, procedures for collection of symptom data were improved following review of symptom data from 2016. These improvements included simplification of the symptom collection form and monthly training of fieldworkers on signs and symptoms identification and recording and frequent emphasis of the importance of symptom reporting to participants.

### 2.4 Laboratory measurements

NP samples were collected using flexible nasopharyngeal nylon flocked swabs (PrimeSwab™, Longhorn Vaccines & Diagnostics, San Antonio, Texas, USA), placed in PrimeStore® Molecular Transport Medium (MTM) (Longhorn Vaccines & Diagnostics, San Antonio, Texas, USA) and transported at 2–8°C to the National Institute for Communicable Diseases (NICD) in Johannesburg, where the samples were tested, aliquoted and stored at 2–8°C before freezing at −70°C. Nucleic acids were extracted from PrimeStore® MTM using the Roche MagNA Pure 96 instrument (Roche, Mannheim, Germany) according to the manufacturer's instructions. NP samples were tested for RSV and influenza A and B viruses by real‐time PCR (RT‐PCR) using the FTD Flu/RSV detection assay (Fast Track Diagnostics, Luxembourg). Influenza A positive samples were subtyped using the Centers for Disease Control and Prevention (CDC) influenza A (H1/H3/H1pdm09) subtyping kit and influenza B lineage was determined using the CDC B/Yamagata‐B/Victoria lineage typing kit (available through International Reagent Resource Program; http://www.internationalreagentresource.org) using SuperScript™ III One‐Step RT‐PCR System with Platinum™ *Taq* DNA Polymerase (ThermoFisher, Waltham, Massachusetts, USA)^20^. RSV A and B subgroups were determined by an in‐house RT‐PCR.^21^, ^22^ Twice‐weekly NP samples were tested for *S. pneumoniae* using an in‐house singleplex (*lytA*) quantitative real‐time PCR assay.^23^ NP and sputum samples were tested for *Bordetella* spp. (including *B. pertussis*, *B. parapertussis*, *B. bronchiseptica* and *B. holmesii*) by a combination of a triplex and singleplex real‐time PCR assays and results interpreted as previously described.^24^

Clotted blood samples from serology surveys collected in vacutainer tubes were centrifuged, aliquoted and stored frozen before being sent in batches to NICD on dry ice. Hemagglutination inhibition (HAI) assays using turkey red blood cells were performed to determine serological reactivity titres for serum samples against influenza.^25^ Virus strains for testing were selected based on the Southern Hemisphere vaccine strains and strains predominantly circulating in South Africa during each year. Cultures from circulating strains representing the following subtypes and lineages were prepared in Madin–Darby Canine Kidney cells: A/Singapore/NFIMH‐16‐0019/2016‐like for influenza A (H3N2), A/Michigan/45/2015‐like for influenza A(H1N1)pdm09, B/Brisbane/60/2008‐like for influenza B Victoria lineage and B/Phuket/3073/2013‐like for influenza B Yamagata lineage. RSV antibody titres were determined using an enzyme‐linked immunosorbent assay (ELISA) (EUROIMMUN Anti‐RSV IgG; Lübeck, Germany).^26^ Serology for pertussis antibody was performed using the commercially available anti‐Pertussis toxin ELISA (EUROIMMUN, Lübeck, Germany).^27^

Urine cotinine tests were performed using the IMMULITE® 1000 Nicotine Metabolite Assay Kit (Siemens Medical Solutions Diagnostics, Gly Rhonwy, UK). Sputum samples were collected at baseline from individuals who could expectorate and thereafter from any individual with fever, cough or weight loss for >2 weeks, assessed monthly. Sputum samples were tested for tuberculosis using GeneXpert MTB/RIF (Cepheid, Sunnyvale, CA, USA) and cultured using the Mycobacteria Growth Indicator Tube (MGIT) 960 instrument (Becton Dickinson, East Rutherford, NJ, USA).

Tuberculin skin tests were performed twice‐annually 6–10 months apart. Five units of purified tuberculin were injected intradermally on the volar aspect of the forearm. Results were assessed 48–72 h thereafter, for induration and, if present, study staff measured the diameter in millimetres transverse to the axis of the forearm. HIV testing, according to the double rapid test algorithm in place in each province was offered to all participants older than 10 years of age.^28^ Participants were considered HIV infected if they had one of the following during the follow‐up period: two positive rapid HIV tests, evidence of a positive HIV laboratory result or evidence of antiretroviral treatment. Participants were considered HIV uninfected if they had a documented negative HIV test result during the study. A documented HIV negative status for the mother was considered confirmation of HIV negative status for a child aged <10 years. Infants were defined as HIV exposed but uninfected if they were HIV uninfected, but the mother was HIV infected. HIV infection was confirmed by PCR in children aged <18 months. For all HIV‐infected individuals, specimens were collected for CD4+ T cell and quantitative HIV viral load testing. Patients newly diagnosed with HIV were referred to the local clinic for assessment and initiation of antiretroviral treatment.

### 2.5 Environmental assessment

Environmental monitoring was conducted in a convenience subset of 150 households for one week each year during summer and winter. Respirable particulate matter <4‐μm diameter (PM_4_) concentrations were measured indoors using a stationary photometric monitor (DustTrak II Model 8530, TSI Incorporated, Shoreview, MN, USA) and gravimetric filter sampling to measure the quantity of airborne particulate matter. During the same period, one member of each household carried a personal exposure monitoring device (SidePak AM510, TSI Incorporated, Shoreview, MN, USA) throughout the daytime. Indoor carbon dioxide and ambient PM_4_ (ES‐642, MetOne Instruments, Inc, Grants Pass, OR, USA) levels were measured during 2018. Thermochron® iButton sensors (Maxim Integrated, San Jose, California, USA) were located in the indoor (all households) and ambient (subset) environments to measure temperature (Model DS1921G‐F5) and relative humidity (Model RS1923l‐F5 in 2018) concurrently with the air pollution monitoring in 2016 and continuously during 2017–2018.

### 2.6 Housing quality survey

Information on housing type, construction, materials and condition, water sources, water security and water storage, fuel use and expenditure for cooking, space and water heating, waste removal services, visible dampness and smoking practices was collected annually for all households.

### 2.7 Proximity and contact study

In 2018, four surveys of household contact using proximity monitors (http://www.sociopatterns.org) were conducted to capture information on intra‐household contact patterns for three seasons (summer, autumn and winter). To measure contacts of participants of the study outside the home, participants were interviewed by field workers to complete a contact diary and time‐use questionnaire for one day between August and October 2018.

### 2.8 Costing survey

We surveyed all symptomatic household members during August–October 2018 to assess cost of medically attended and non‐medically attended illness episodes.

## 3 FINDINGS TO DATE

### 3.1 Household and individual characteristics

**FIGURE 3:**
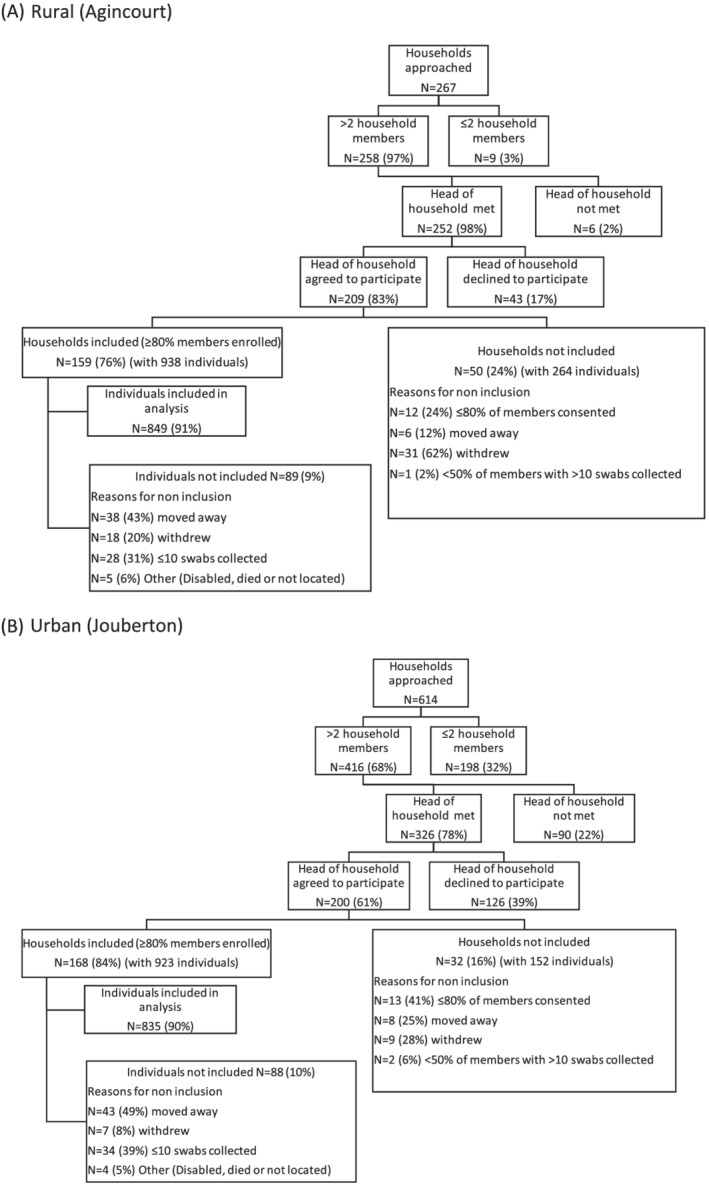
Flow chart of participant enrolment at the rural and urban sites, South Africa, 2016–2018. (A) Rural (Agincourt) and (B) urban (Jouberton). Individuals or households were not included if they withdrew or moved away before completing >10 visits with collection of a nasopharyngeal swab. Bars indicate timing of serology blood draws. Grey bars indicate number of nasopharyngeal swabs tested each visit. Blood draw dates in year **2016** were Draw 1 (16 March to 15 April) and Draw 2 (17 October to 24 November). Blood draw dates in year **2017** were 2016 cohort Draw 1 (6 February to 4 March), Draw 2 (3–26 May), Draw 3 (2–18 October); 2017 cohort Draw 1 (24 November 2016 to 24 February 2017), Draw 2 (2–26 May) and Draw 3 (12–27 October). Blood draw dates in year **2018** were 2016 cohort Draw 1 (29 January to 9 February), Draw 2 (4 May to 6 June), Draw 3 (1 October to 5 November); 2017 cohort Draw 1 (12 February to 9 March), Draw 2 (4 May to 4 June), Draw 3 (17–31 October); 2018 cohort Draw 1 (28 November 2017 to 24 January 2018), Draw 2 (21 April to 1 June 2018) and Draw 3 (1–31 October)

From 2016 to 2018 at both sites combined, 881 households were approached: for 409 (46%) households, the head of household agreed to participate and 327 (78%) of these were included in the final analysis (Figures 3a and 2b). There were 1861 individuals residing in the 327 included households, of whom, 1684 (90%) consented and were included in the final analysis. Reasons for non‐inclusion are shown in Figure 3a,b. A higher percentage of approached houses were included at the rural site (159/267, 60%) compared to the urban site (168/614, 27%). This is because rural site houses with >2 members were pre‐selected from the HDSS database and a higher proportion of household members consented to participate in the study among eligible households (209/252, 83% vs. 200/326, 61%, *p* < .001).

At the rural site, characteristics of included households were similar to those of households within the HDSS that were not included (Table 2a). However, when compared to individuals from the HDSS who were not included, included individuals were less likely to be aged 15–44 years, male, employed or have completed secondary education. This likely reflects migrant worker patterns and the fact that within included households, males and individuals aged 15–44 years were less likely to participate in the study (Table S2). At the urban site, characteristics of households and individuals included in PHIRST were compared to those of residents of Jouberton Township from the 2011 census^29^ (Table 2b). Compared to the general population, included households were more likely to be formal (brick houses on a municipal stand) houses rather than shacks within informal settlements, possibly because larger stands are more likely to be sampled using GPS coordinates. HIV prevalence among included individuals aged 15–49 years with available data was 27% (99/370) at the rural site and 29% (97/329) at the urban site, compared to 23% (95% CI 20–26%) and 23% (95% CI 18–28) reported for Mpumalanga (rural site) and North West (urban site) Provinces in 2017, respectively.^30^

**TABLE 2a:** Characteristics of included participants and households in PHIRST during 2016–2018 at the rural site (Agincourt) compared to those not included, using data from the 2017 Agincourt health and socio‐demographic surveillance system site (HDSS) census

Characteristic	Included *n*/*N* (%) or number (range)	Not included *n*/*N* (%) or number (range)	*p*
Household level			
Mean number (range) members in household	4 (1–20)	4 (1–15)	.479
Modern house (vs. traditional)	136/136 (100)	16 738/16 785(99.7)	.587
Toilet site			
In house	2/136 (1)	268/16 782 (2)	.658
In yard	121/136 (89)	15 249/16 782 (91)	
Other	13/136 (10)	1265/16 782 (8)	
Type of toilet			
Modern	3/136 (2)	245/16 780 (1)	.151
VIP	7/136 (5)	1841/16 780 (11)	
Pit latrine	116/136 (85)	13 676/16 780 (82)	
Other	10/136 (7)	1018/16 780 (6)	
Source of energy for cooking			
Electricity	58/136 (43)	8929/16 781 (53)	.050
Wood	77/136 (57)	7759/16 781 (46)	
Gas/Paraffin (kerosene)/Other	1/136 (1)	93/16 781 (1)	
Individual level			
Age group (years)			
<1	2/791 (0.3)	1302/99 645 (1)	<.001
1–4	114/791 (14)	9079/99 645 (9)	
5–14	296/791 (37)	22 568/99 645 (23)	
15–44	274/791 (35)	51 128/99 645 (51)	
45–64	70/791 (9)	9966/99 645 (10)	
65+	35/791 (4)	4602/99 645 (5)	
Female sex	499/791 (63)	52 972/98 640 (54)	<.001
Level of education^a^			
None	173/704 (25)	17 500/88 149 (20)	<.001
Primary	265/704 (38)	24 238/88 149 (27)	
Some secondary	202/704 (29)	26 088/88 149 (30)	
Completed secondary	64/704 (9)	20 322/88 149 (23)	
Post‐secondary	0/704 (0)	1/88 149	
Currently working^b^	60/495 (12)	19 267/74 923 (26)	<.001

*Note*: Only households and individuals with data available in the Agincourt HDSS included, for individual level analysis only permanent (non‐migrant) household members included. Data are mean (range) or *n*/*N* (%).

Abbreviation: VIP, ventilated improved pit latrine.

^a^ The highest level an individual has attained at the time of observation.

^b^ Individuals aged ≥17 years.

**TABLE 2b:** Characteristics of included participants at the urban site (Jouberton) during 2016–2018 from PHIRST database and characteristics of the general population from the 2011 Census^29^

Characteristic	Enrolled *n*/*N* (%) or number (range)	General population^a^ *n*/*N* (%) or number (range)

Household level		
Mean number (range) members in household	5 (3–14)	4 (3–5)^b^
Mean number (range) rooms in household	5 (2–11)	4 (1–18)
Monthly household income		
≤R800 (<$54)	18/167 (11)	7119/32 136 (22)
R801–R1600 ($55–$108)	35/167 (21)	2835/32 136 (9)
R1601–R3200 ($109–$116)	49/167 (29)	6507/32 136 (20)
R3201–R6400 ($117–$232)	34/167 (20)	7251/32 136 (23)
R6401–R12800 ($233–$464)	9/167 (5)	4851/32 136 (15)
>R12800 (>$464)	5/167 (3)	3558/32 136 (11)
Did not disclose	17/167 (10)	15/32 136 (0)
Type of dwelling		
Formal house	158/167 (95)	23 379/32 130 (73)
Informal dwelling	6/167 (4)	6777/32 130 (21)
Formal dwelling in backyard	0/167 (0)	882/32 130 (3)
Flat	0/167 (0)	225/32 130 (1)
Traditional dwelling	0/167 (0)	93/32 130 (0)
Other	3/167 (2)	774/32 130 (2)
Individual level		
Age group (years)		
<1	27/858 (3)	2664/111 936 (2)
1–4	89/858 (10)	10 590/111 936 (9)
5–14	238/858 (28)	21 051/111 936 (19)
15–44	334/858 (39)	54 297/111 936 (49)
45–64	124/858 (14)	18 684/111 936 (17)
65+	46/858 (5)	4650/111 936 (4)
Female sex	487/859 (57)	57 129/111 939 (51)
Level of education^c^		
No schooling	26/443 (6)	7872/98 442 (8)
Primary schooling	116/443 (26)	34 767/98 442 (35)
Some secondary	207/443 (47)	35 295/98 442 (36)
Secondary completed	81/443 (18)	18 015/98 442 (18)
Post‐secondary	13/443 (3)	2493/98 442 (3)
Currently working^d^	154/444 (35)	24 642/72 981 (34)

^a^ Data on general population obtained from census 2011^29^ unless otherwise indicated. Data are median (IQR) or *n*/*N* (%).

^b^ Source: Wong et al.^18^

^c^ >18 years PHIRST, >5 years in census.

^d^ >18 years PHIRST, >15 years in census.

Among 327 households included in the study, the median household size was five individuals (interquartile range 3–10) and 160 (49%) reported crowding (>2 people per sleeping room) (Table 3). Among 1684 study participants, 16% were aged <5 years, 32% aged 5–14 years and 60% were female. Compared to the urban site, households in the rural site were more likely to have a child aged <5 years in the house and to use wood as fuel for cooking, and less likely to report smoking in the house or to have a handwashing place with water. Compared to the urban site, individuals in the rural site were more likely to be aged <15 years, female, unemployed and less likely to drink alcohol or smoke.

**TABLE 3:** Baseline characteristics of households and participants included in the final cohort by site, a rural and an urban site in South Africa, PHIRST study, 2016–2018

Characteristic	Overall *n* (%) or median (IQR)	Rural *n* (%) or median (IQR)	Urban *n* (%) or median (IQR)	OR (95% CI)	*p*
Household level characteristics	*N* = 327	*N* = 159	*N* = 168		
Year					
2016	100 (31)	50 (31)	50 (30)	Reference	.932
2017	109 (33)	53 (33)	56 (33)	1.1 (0.6–1.8)	
2018	118 (36)	56 (35)	62 (37)	1.1 (0.6–1.9)	
Number of household members					
3–5	196 (60)	89 (56)	107 (64)	Reference	.341
6–10	119 (36)	63 (40)	56 (33)	0.7 (0.5–1.2)	
>10	12 (4)	7 (4)	5 (3)	0.6 (0.2–1.9)	
Number of household members	5 (3–10)	5 (3–11)	5 (3–10)	Not estimated	.442
Number of rooms	5 (2–9)	5 (1–10)	5 (2–8)	Not estimated	.453
Number of rooms for sleeping	2 (1–4)	3 (1–4)	2 (1–4)	Not estimated	.256
Crowding (>2 people per sleeping room)	160 (49)	83 (52)	77 (46)	0.8 (0.5–1.2)	.250
Child aged <5 years in house	225 (69)	141 (89)	84 (50)	0.1 (0.1–0.2)	<.001
HIV‐infected household member	172 (53)	80 (50)	92 (55)	1.2 (0.8–1.8)	.421
Cigarette smoke in house	71 (22)	18 (11)	53 (32)	3.6 (2.0–6.5)	<.001
Main water source tap inside (vs. tap outside)	154 (47)	71 (45)	83 (49)	0.8 (0.5–1.3)	.390
Handwashing place with water in house	264 (81)	100 (63)	164 (98)	24.2 (8.6–68.3)	<.001
Main fuel for cooking					
Electricity	244 (75)	89 (56)	155 (93)	121.9 (16.6–892.8)	<.001
Wood	71 (22)	70 (44)	1 (1)	Reference	
Paraffin (kerosene)/gas/other	10 (3)	0 (0)	10 (6)	Not estimated	
Monthly household income^a^					
≤R800 (<$54)	39 (12)	21 (14)	18 (11)	Reference	.807
R801–R1600 ($55–$108)	75 (24)	36 (23)	39 (24)	1.3 (0.6–2.7)	
R1601–R3200 ($109‐$116)	109 (34)	56 (36)	53 (33)	1.1 (0.5–2.3)	
R3201–R6400 ($117–$232)	70 (22)	32 (21)	38 (23)	1.4 (0.6–3.0)	
R6401–R12800 ($233–$464)	18 (6)	8 (5)	10 (6)	1.5 (0.5–4.5)	
>R12800 (>$464)	7 (2)	2 (1)	5 (3)	0.9 (0.5–16.8)	
Individual level characteristics	*N* = 1684	*N* = 849	*N* = 835		
Age group (years)					
<1	36 (2)	15 (2)	21 (3)	2.5 (1.2–5.1)	<.001
1–4	243 (14)	156 (18)	87 (10)	Reference	
5–14	547 (32)	309 (36)	238 (29)	1.4 (1.1–1.9)	
15–44	590 (35)	265 (31)	325 (39)	2.1 (1.6–3.0)	
45–64	195 (12)	74 (9)	121 (14)	2.9 (2.0–4.3)	
65+	73 (4)	30 (4)	43 (5)	2.6 (1.5–4.4)	
Female sex	1009 (60)	533 (63)	476 (57)	0.8 (0.6–0.9)	.016
Year					
2016	542 (32)	280 (33)	262 (31)	Reference	.768
2017	577 (34)	289 (34)	288 (34)	1.1 (0.8–1.3)	
2018	565 (34)	280 (33)	285 (34)	1.1 (0.9–1.4)	
Level of education^b^					
No schooling	90 (12)	65 (22)	25 (6)	Reference	<.001
Primary schooling	158 (22)	60 (20)	98 (23)	4.2 (2.4–7.5)	
Some secondary	285 (39)	85 (28)	200 (47)	6.1 (3.6–10.3)	
Secondary completed	174 (24)	87 (29)	87 (21)	2.6 (1.5–4.5)	
Post‐secondary	19 (3)	5 (2)	14 (3)	7.3 (2.4–22.3)	
Employment^b^					
Unemployed	400 (55)	182 (60)	218 (51)	Reference	.014
Employed	267 (37)	88 (29)	179 (42)	1.7 (1.2–2.3)	
Student	59 (8)	32 (11)	27 (6)	0.7 (0.9–1.5)	
Reported alcohol use^c^	337 (39)	57 (15)	280 (57)	7.3 (5.2–10.2)	<.001
Reported current cigarette smoking^c^	139 (16)	22 (6)	117 (24)	4.9 (3.0–7.9)	<.001
Reported current snuff smoking^c^	97 (11)	5 (1)	92 (19)	17.0 (6.8–42.1)	<.001
Reported current any smoking^c^	242 (28)	28 (8)	214 (44)	9.5 (6.2–14.4)	<.001
Cigarette smoke inside house^d^	99 (41)	7 (25)	92 (43)	2.3 (0.9–5.5)	.048
Urine cotinine (all ages)^e^					
Negative	649 (42)	530 (68)	119 (16)	Reference	<.001
Passive exposure	660 (43)	222 (28)	438 (57)	8.8 (6.8–11.4)	
Active smoking	241 (16)	31 (4)	210 (27)	30.2 (19.7–46.2)	
Unknown	134	66	68	Not included	
HIV status^e^					
Uninfected	1379 (85)	715 (86)	664 (83)	Reference	.158
Infected	249 (15)	117 (14)	132 (17)	1.2 (0.9–1.6)	
Unknown	56	17	39	Not included	
Previous tuberculosis	88 (5)	15 (2)	73 (9)	5.3 (3.0–9.4)	<.001
Current tuberculosis	24 (1)	3 (<1)	21 (3)	7.3 (2.2–24.5)	<.001
Other underlying illness^f^	50 (3)	5 (1)	45 (5)	9.6 (3.8–24.3)	<.001
Influenza vaccination current year	2 (<1)	1 (<1)	1 (<1)	1.0 (0.1–16.2)	.990
Pneumococcal vaccine up to date for age^g^					
Yes	221 (81)	137 (81)	84 (80)	0.2 (0.1–1.3)	.182
No	7 (3)	2 (1)	5 (5)	Reference	
No data	46 (17)	30 (18)	16 (15)	0.2 (0.1–1.2)	
DTaP‐IPV/Hib vaccine up to date for age^g^					
Yes	222 (81)	135 (80)	87 (83)	0.9 (0.2–3.9)	.735
No	7 (3)	4 (2)	3 (3)	Reference	
No data	45 (16)	30 (18)	15 (14)	0.7 (0.1–3.4)	

^a^ Data available for 318 households, 155 rural and 163 urban.

^b^ Individuals aged ≥18 years *N* = 726, 302 at rural and 424 at urban site.

^c^ Self‐reported, individuals aged ≥15 years *N* = 858, 369 at rural and 489 at urban site.

^d^ Amongst those reporting any current smoking.

^e^ % and *p* value among individuals with known status.

^f^ Self‐reported history of asthma, lung disease, heart disease, stroke, spinal cord injury, epilepsy, organ transplant, immunosuppressive therapy, organ transplantation, cancer, liver disease, renal disease or diabetes.

^g^ Individuals aged <5 years *N* = 274, 169 at rural site and 105 at urban site, 229 with available vaccination data, 139 at the rural site and 90 at the urban site.

Among 1684 included individuals, 1605 (95%) were present at end of the twice‐weekly follow‐up phase. Of 79 lost to follow‐up, 53 (67%) left the study sites, 21 (27%) withdrew and 5 (6%) died (Table 4). Just over half of the individuals in the 2016 and 2017 cohorts completed three serology blood draws in the years after completion of the swabbing phase. Individuals lost to follow‐up were more likely to be aged 15–44 years, possibly due to economic migration, compared to those completing follow‐up (Table S3).

**TABLE 4:** Follow‐up rates by site and year among 1684 individuals included in the analysis of the PHIRST study, South Africa, 2016–2018

		Site		Year		
Characteristic	Overall *N* (%)	Rural *N* (%)	Urban *N* (%)	2016 *N* (%)	2017 *N* (%)	2018 *N* (%)
Twice‐weekly follow‐up phase						
Individuals						
Total included in analysis	1684	849	835	542	577	565
Initially enrolled^a^	1555 (92)	790 (93)	765 (92)	505 (93)	551 (95)	499 (88)
Late enrolments for replacement or in‐migration to household	129 (8)	59 (7)	70 (8)	37 (7)	26 (5)	66 (12)
						
Lost to follow‐up	79 (5)	32 (4)	47 (6)	21 (4)	31 (5)	27 (5)
Withdrawal n(%)	21 (27)	13 (41)	8 (17)	9 (43)	6 (19)	6 (22)
Left study site n(%)	53 (67)	17 (53)	36 (77)	12 (57)	21 (68)	20 (74)
Death n(%)	5 (6)	2 (6)	3 (6)	0 (0)	4 (13)	1 (4)
Completed follow‐up	1605 (95)	817 (96)	788 (94)	521 (96)	546 (95)	538 (95)
Households						
Total included	327	159	168	100	109	118
Initially enrolled^a^	308 (94)	148 (93)	160 (95)	96 (96)	105 (96)	107 (91)
Late enrolments for replacement or in‐migration to household	19 (6)	11 (7)	8 (5)	4 (4)	4 (4)	11 (9)
						
Lost to follow‐up	6 (2)	1 (1)	5 (3)	1 (1)	2 (2)	3 (3)
Withdrawal *n* (%)	3 (1)	1 (1)	2 (1)	1 (1)	2 (2)	0 (0)
Left study site *n* (%)	3 (1)	0 (0)	3 (2)	0 (0)	0 (0)	3 (3)
Completed follow‐up	321 (98)	158 (99)	163 (97)	99 (99)	107 (98)	115 (97)
Serology phase individuals^b^	*n*/*N* (%)	*n*/*N* (%)	*n*/*N* (%)			
2016 cohort: three blood draws in serology phase 2017	297/542 (55)	116/280 (41)	181/262 (69)	NA	NA	NA
2016 cohort: three blood draws in serology phase 2018	295/542 (54)	117/280 (42)	178/262 (68)	NA	NA	NA
2017 cohort: three blood draws in serology phase 2018	303/577 (53)	118/289 (41)	185/288 (64)	NA	NA	NA

Abbreviation: NA, not applicable.

^a^ Before 31 March for 2016, before 31 January for 2017 and 2018.

^b^ Timing of blood draws indicated graphically in Figure 3. Participants in the 2016 cohort had two blood draws during 2016 and three each in 2017 and 2018, participants in the 2017 cohort had three blood draws in 2017 and three in 2018, participants in the 2018 cohort had three blood draws in 2018.

### 3.2 Samples collected and symptoms

There were 122 113 potential individual follow‐up visits over the 3 years, and participants were interviewed for 105 783 (87%) of these. In 2017–2018, there were 94 786 potential visits, of which participants were interviewed for 81 943 (86%) and 81 928 (>99%) had available data on symptoms. At least one symptom was reported for 8% (6692) of visits overall. At least one symptom over the follow‐up period in 2017–2018 was reported by 89% (1012/1142) of individuals and was more commonly reported among children aged <5 years (97%, 180/185) compared to older individuals (5–18 years 87%, 404/466; 19–65 years 87%, 390/450; >65 years 93%, 38/41, *p* < .001 chi‐squared test). The commonest symptoms reported were cough (76%, 863/1142) and runny nose (74%, 841/1142). The rate of clinic visits in 2017–2018 for acute complaints was 1.3 per 100 person weeks of follow‐up (*n* = 520) (2.1 in <5 years, 0.9 in 5–18 years, 1.3 in 19–65 years and 2.0 in >65 years) and of hospitalisations was 0.05 per 100 person weeks of follow‐up (*n* = 36) (0.09 in <5 years, 0.05 in 5–18 years, 0.10 in 19–65 years and 0.3 in >65 years).

From May 2016 to December 2018, a total of 105 683 nasopharyngeal swabs, 4217 clotted blood samples, 1442 whole blood samples, 1567 urine samples and 741 sputum samples were collected. Out of 105 683 nasopharyngeal swabs from follow‐up visits collected and tested from 1684 participants, in 327 households, 1258 (1%), 1026 (1%), 273 (<1%), 38 829 (37%) tested positive on PCR for influenza viruses, RSV, pertussis and pneumococcus, respectively.

Analysis of data from 2017–2018 on influenza identified high attack rates with 79% of households had at least one individual testing influenza positive each season and 37% of household members infected at least once with PCR‐confirmed influenza each year.^31^ Incidence was similar in the urban and rural site. Repeat influenza infections within the same season were identified in 17% of individuals experiencing at least one influenza infection and were more common in children. The incidence of PCR‐confirmed influenza infection was highest among children aged <5 years and decreased with increasing age. Overall, 56% of infections were associated with ≥1 symptom and 35% of these had fever and cough. The proportion of symptomatic infections was higher in children aged <5 years (74% in this age group vs. 39% in those aged 19–44 years). Overall, there was influenza transmission to 10% of household contacts of an index case. Transmission was highest among children and individuals with ≥2 symptoms (17%); however, asymptomatic individuals did transmit influenza to 6% of household contacts.

### 3.3 Future plans

Households enrolled in the PHIRST study during 2016–2018 were eligible for inclusion in a study of SARS‐CoV‐2 transmission initiated in July 2020. This study uses similar testing frequency and household selection methods to assess the community burden of SARS‐CoV‐2 infection and the role of asymptomatic infection in virus transmission.

## 4 STRENGTHS AND LIMITATIONS

### 4.1 Strengths

PHIRST was conducted in urban and rural African settings. Situation in a high HIV prevalence setting with high study uptake of HIV testing (97%) allows assessment of the effect of HIV on community burden and transmission dynamics of respiratory pathogens. Households were selected randomly to provide a representative sample of the community. Sampling from individuals irrespective of symptoms allows estimation of community burden, household secondary infection risk and serial interval including asymptomatic or paucisymptomatic episodes.^32^ It also allows the estimation of the proportion of transmission from asymptomatic individuals. PHIRST utilised multiple laboratory‐confirmed infection endpoints including PCR and serology, which provide additional data on the community burden of these pathogens and allows evaluation of the correlation between pathogen detection and serological response in individuals of different age and HIV‐infection status. Laboratory confirmation of multiple respiratory pathogens simultaneously allows study of the effect of respiratory co‐infections on disease severity and transmissibility and the interaction between different pathogens. Our twice‐weekly sampling strategy was unlikely to miss many episodes of infection and allowed accurate ascertainment of first and subsequent infections from the same or different pathogens in the household. Our long period of active follow‐up for each cohort allowed us to describe burden and transmission of infection within described seasons.

### 4.2 Limitations

This study was not powered to assess severe outcomes (i.e., hospitalisation and death). Repeated assessment of symptoms at twice‐weekly visits over an extended period may lead to possible fatigue and under‐reporting by participants. High rates of migration and movement in communities under study affected follow‐up rates. The study was intensive, and nasopharyngeal swabs are uncomfortable for participants, which may have resulted in fewer participants consenting and reduced follow‐up rates. Quality of specimen collection may have varied; however, >99% of samples tested positive for the presence of human DNA. We only examined four pathogens, but other micro‐organisms may be important. Samples have been stored which could allow us to implement broader multi‐pathogen testing in the future. We did not test staff members for the presence of organisms under study, because a previous similar study in which staff were sampled weekly did not identify any influenza or RSV infections among field staff.^33^

## ETHICAL REVIEW

The protocol was registered on http://clinicaltrials.gov on 6 August 2015 (Reference NCT02519803) and was approved by the University of the Witwatersrand Human Research Ethics Committee (Reference 150808) and the US CDC's Institutional Review Board relied on the local review (#6840). Participants provided individual written consent or assent prior to enrolment and received a grocery store voucher of ZAR25‐30 (USD 2–2.5) per visit for their time.

## PATIENT AND PUBLIC INVOLVEMENT

Both study sites have community advisory boards (CAB) consisting of representatives from community‐based and faith‐based organisations who were involved in the planning of the PHIRST study. The CABs meet regularly and give advice on protocols, consents and recruitment plans and also provide feedback to communities on results of studies. In addition, feedback sessions on study findings were held for participating families.

## CONFLICT OF INTEREST

Cheryl Cohen has received research grants awarded to her institution from Sanofi Pasteur, US Centers for Disease Control and Prevention. Cheryl Cohen has had costs for travel to a meeting supported by Parexel. Maimuna Carrim was awarded the Robert Austrian Research Award in Pneumococcal Vaccinology sponsored by Pfizer. Neil Martinson has a research grant awarded to his institution by Pfizer South Africa. Anne von Gottberg has received research grants awarded to her institution from Sanofi Pasteur, Pfizer and US Centers for Disease Control and Prevention.

## AUTHOR CONTRIBUTIONS

CC, MM, TM, JM, FKT, OH, NW, NAM, KK, AvG and ST: conception or design of protocol. All co‐authors: acquisition, analysis or interpretation of data for the work. FKT, OH, NW, MC, AB, LM and AvG: laboratory testing of samples. LL, MM, KM, FW and SN: enrolment and follow‐up of participants. CC, MM and ST: drafting the work or revising it critically for important intellectual content. All co‐authors: final approval of the version to be published. All co‐authors: agreement to be accountable for all aspects of the work in ensuring that questions related to the accuracy or integrity of any part of the work are appropriately investigated and resolved.

## DISCLAIMER

The findings and conclusions in this report are those of the author(s) and do not necessarily represent the official position of the funding agencies.

### PEER REVIEW

The peer review history for this article is available at https://publons.com/publon/10.1111/irv.12881.

## Supporting information


**Table S1** PHIRST study primary and secondary objectives and public health importance of questions
**Table S2** Comparison of characteristics of individuals who participated in the study compared to those who did not participate within included households, a rural and an urban site, South Africa, 2016–2018
**Table S3** Comparison of individual and household‐level characteristics of individuals lost to follow‐up for twice‐weekly visits to those with complete follow‐up, a rural and an urban site, South Africa, 2016–2018
**Figure S1** Timing of study procedures in relation to enrolment, follow‐up and season, a rural and an urban site, South Africa, 2016–2018 (A) rural site and (B) urban site

## Data Availability

The study protocol including informed consent forms is available on the NICD website (https://www.nicd.ac.za/wp-content/uploads/2021/02/PHIRST-SARS-CoV-2-protocol-V1-amendment-Nov2020-incl-upd-consent.pdf). Analysis of the data for primary study objectives is planned to be completed by December 2023. Additional modelling and serologic studies will be concluded within one additional year, and primary de‐identified data will be made publicly available not later than December 2025. The investigators welcome enquiries about possible collaborations and requests for access to the data set. Data will be shared after approval of a proposal and with a signed data access agreement. Investigators interested in more details about this study, or in accessing these resources, should contact the principle investigator, Prof Cheryl Cohen, at NICD (cherylc@nicd.ac.za).

## References

[1] Troeger C , Forouzanfar M , Rao PC , et al. Estimates of the global, regional, and national morbidity, mortality, and aetiologies of lower respiratory tract infections in 195 countries: a systematic analysis for the Global Burden of Disease Study 2015. Lancet Infect Dis. 2017;17(11):1133‐1161. 10.1016/S1473-3099(17)30396-1 28843578 PMC5666185

[2] Iuliano AD , Roguski KM , Chang HH , et al. Estimates of global seasonal influenza‐associated respiratory mortality: a modelling study. Lancet. 2017;391(10127):1285‐1300. 10.1016/S0140-6736(17)33293-2 29248255 PMC5935243

[3] Roth GA , Abate D , Abate KH , et al. Global, regional, and national age‐sex‐specific mortality for 282 causes of death in 195 countries and territories, 1980–2017: a systematic analysis for the Global Burden of Disease Study 2017. Lancet. 2018;392(10159):1736‐1788. 10.1016/S0140-6736(18)32203-7 30496103 PMC6227606

[4] Shi T , McAllister DA , O'Brien KL , et al. Global, regional, and national disease burden estimates of acute lower respiratory infections due to respiratory syncytial virus in young children in 2015: a systematic review and modelling study. Lancet. 2017;390(10098). 10.1016/S0140-6736(17)30938-8 PMC5592248 28689664

[5] Wahl B , Brien KLO , Greenbaum A , et al. Articles Burden of *Streptococcus pneumoniae* and *Haemophilus influenzae* type b disease in children in the era of conjugate vaccines: global, regional, and national estimates for 2010–15. Lancet Glob Heal. 2018;6(7):e744‐e757. 10.1016/S2214-109X(18)30247-X PMC6005122 29903376

[6] Tsang TK , Lau LL , Cauchemez S , Cowling BJ . Household transmission of influenza virus. Trends Microbiol. 2015;24(2):123‐133. 10.1016/j.tim.2015.10.012 26612500 PMC4733423

[7] Kombe IK , Munywoki PK , Baguelin M , Nokes DJ , Medley GF . Model‐based estimates of transmission of respiratory syncytial virus within households. Epidemics. December 2018;27:1‐11. 10.1016/j.epidem.2018.12.001 30591267 PMC6543068

[8] Whitney CG , Goldblatt D , O'Brien KL . Dosing schedules for pneumococcal conjugate vaccine. Pediatr Infect Dis J. 2014;33(SUPPL. 2):172‐181. 10.1097/INF.0000000000000076 PMC3940379 24336059

[9] WHO . Pertussis vaccines: WHO position paper. Wkly Epidemiol Rec. 2010;40(85):385‐400. 20939150

[10] Pebody RG , Sinnathamby MA , Warburton F , et al. Uptake and impact of vaccinating primary school‐age children against influenza: experiences of a live attenuated influenza vaccine programme, England, 2015/16. Eurosurveillance. 2018;23(25):pii=1700496. 10.2807/1560-7917.ES.2018.23.25.1700496 PMC6152241 29945698

[11] Munywoki PK , Koech DC , Agoti CN , et al. The source of respiratory syncytial virus infection in infants: a household cohort study in rural Kenya. J Infect Dis. 2014;209(11):1685‐1692. 10.1093/infdis/jit828 24367040 PMC4017365

[12] Fragaszy EB , Warren‐Gash C , White PJ , et al. Effects of seasonal and pandemic influenza on health‐related quality of life, work and school absence in England: Results from the Flu Watch cohort study. Influenza Other Respi Viruses. 2018;12(1):171‐182. 10.1111/irv.12506 PMC5818341 28991409

[13] Statistics South Africa . Investigation into appropriate definitions of urban and rural areas for South Africa discussion document. Pretoria, South Africa; 2001.

[14] Cohen C , Walaza S , Moyes J , et al. Epidemiology of viral‐associated acute lower respiratory tract infection among children <5 years of age in a high HIV prevalence setting, South Africa, 2009–2012. Pediatr Infect Dis J. 2015;34(1):66‐72. 10.1097/INF.0000000000000478 25093972 PMC4276570

[15] Kahn K , Collinson MA , Xavier Gómez‐olivé F , et al. Profile: Agincourt health and socio‐demographic surveillance system. Int J Epidemiol. 2012;41(4):988‐1001. 10.1093/ije/dys115 22933647 PMC3429877

[16] Garenne M , Collinson MA , Kabudula CW , Gómez‐Olivé FX , Kahn K , Tollman S . Completeness of birth and death registration in a rural area of South Africa: the Agincourt health and demographic surveillance, 1992–2014. Glob Health Action. 2016;9(1):32795. 10.3402/gha.v9.32795 27782873 PMC5081031

[17] World Bank . The World Bank. Overview of South Africa. http://www.worldbank.org/en/country/southafrica/research. 2016.

[18] Wong KK‐L , von Mollendorf C , Martinson N , et al. Healthcare utilization for common infectious disease syndromes in Soweto and Klerksdorp, South Africa. Pan Afr Med J. 2018;30(271). 10.11604/pamj.2018.30.271.14477 PMC6317391 30637056

[19] Harris PA , Taylor R , Minor BL , et al. The REDCap consortium: building an international community of software platform partners. J Biomed Inform. 2019;95:103208. 10.1016/j.jbi.2019.103208 31078660 PMC7254481

[20] Jernigan DB , Lindstrom SL , Johnson JR , et al. Detecting 2009 pandemic influenza A (H1N1) virus infection: availability of diagnostic testing led to rapid pandemic response. Clin Infect Dis. 2011;52 Suppl 1(suppl_1):S36–S43. 10.1093/cid/ciq020 21342897

[21] Hu A , Colella M , Tam JS , Rappaport R , Cheng S‐M . Simultaneous detection, subgrouping, and quantitation of respiratory syncytial virus A and B by real‐time PCR. J Clin Microbiol. 2003;41(1):149‐154. 10.1128/jcm.41.1.149-154.2003 12517840 PMC149607

[22] van de Pol AC , Wolfs TFW , van Loon AM , et al. Molecular quantification of respiratory syncytial virus in respiratory samples: reliable detection during the initial phase of infection. J Clin Microbiol. 2010;48(10):3569‐3574. 10.1128/JCM.00097-10 20660210 PMC2953131

[23] Carvalho MG , Tondella ML , McCaustland K , et al. Evaluation and improvement of real‐time PCR assays targeting lytA, ply, and psaA genes for detection of pneumococcal DNA. JClinMicrobiol. 2007;45(8):2460‐2466. 10.1128/JCM.02498-06 PMC1951257 17537936

[24] Tatti KM , Sparks KN , Boney KO , Tondella ML . Novel multitarget real‐time PCR assay for rapid detection of Bordetella species in clinical specimens. J Clin Microbiol. 2011;49(12):4059‐4066. 10.1128/JCM.00601-11 21940464 PMC3232951

[25] World Health Organisation . Manual for the Laboratory Diagnosis and Virological Surveillance of Influenza. Serological Diagnosis of Influenza by Haemagglutination Inhibition Testing; 2011.

[26] Hacimustafaoglu M , Celebi S , Aynaci E , et al. The progression of maternal RSV antibodies in the offspring. Arch Dis Child. 2004;89(1):52‐53. 10.1136/adc.2002.017780 14709507 PMC1755925

[27] Wu D‐X , Chen Q , Yao K‐H , et al. Pertussis detection in children with cough of any duration. BMC Pediatr. 2019;19(1):236. 10.1186/s12887-019-1615-3 31299934 PMC6626350

[28] National Department of Health: South African Ministry of H . National Consolidated Guidelines for the Prevention of Mother to Child Transmission of HIV (PMTCT) and the Management of HIV in Adolescents and Adults; 2015.

[29] Statistics South A . Census 2011 Statistical release—P0301.4. Stat South Africa. 2012.

[30] Human Sciences Research Council (HSRC) . The Fifth South African National HIV Prevalence, Incidence, Behaviour and Communication Survey, 2017: HIV Impact Assessment Summary Report. Cape Town, South Africa; 2018. http://www.hsrc.ac.za/uploads/pageContent/9234/SABSSMV_Impact_Assessment_Summary_ZA_ADS_cleared_PDFA4.pdf

[31] Cohen C , Kleynhans J , Moyes J , et al. Asymptomatic transmission and high community burden of seasonal influenza in an urban and a rural community in South Africa, 2017–18 (PHIRST): a population cohort study. Lancet Glob Health. 2021;9(6):e863–e874. 10.1016/s2214-109x(21)00141-8 34019838 PMC8262603

[32] Fox J , Brandt C , Wassermann F , et al. The virus watch program: a continuing surveillance of viral infections in Metropolitan New York families. Am J Epidemiol. 1969;80(1):25‐50. 10.1093/oxfordjournals.aje.a120913 4303049

[33] Munywoki PK . Transmission of respiratory syncytial virus in households: who acquires infection from whom?. 2013. oro.open.ac.uk.

