# Supplementary material for: Cohort profile: A Prospective Household cohort study of Influenza, Respiratory syncytial virus and other respiratory pathogens community burden and Transmission dynamics in South Africa, 2016–2018
Source: Influenza Other Respir Viruses. 2021 Jul 23;15(6):789–803. doi: 10.1111/irv.12881 (PMC8542945; doi:10.1111/irv.12881)
Supplement: Supplementary file 1 — Table S1 PHIRST study primary and secondary objectives and public health importance of questions Table S2 Comparison of characteristics of individuals who participated in the study compared to those who did not participate within included households, a rural and an urban site, South Africa, 2016–2018 Table S3 Comparison of individual and household‐level characteristics of individuals lost to follow‐up for twice‐weekly visits to those with complete follow‐up, a rural and an urban site, South Africa, 2016–2018 Figure S1 Timing of study procedures in relation to enrolment, follow‐up and season, a rural and an urban site, South Africa, 2016–2018 (A) rural site and (B) urban site [file IRV-15-789-s001.docx]

**Supplementary tables and figures**

Supplementary table 1: PHIRST study primary and secondary objectives and public health importance of questions

| **Type of objective** | **Objective** | **Public health importance** |
| --- | --- | --- |
| Primary objectives | 1. To estimate the community burden of influenza and RSV, including:    1. the incidence of influenza and RSV infection in the community as determined by polymerase chain reaction (PCR) and serologic assays;    2. the symptomatic fraction associated with influenza and RSV infection;    3. the severity associated with symptomatic infections; and    4. the fraction of individuals with symptomatic infection seeking medical care 2. To assess the transmission dynamics of influenza and RSV infections in the community, including:    1. the estimation of the household secondary infection risk (SIR), serial interval and length of shedding;    2. the estimation of transmission of infection between individuals (both symptomatic and symptomatic) within the household and possibly the community; and    3. the estimation of the effective reproductive number (R) and its variation over time in the community. | In South Africa, influenza vaccination is recommended for individuals at high risk of severe outcomes although coverage is low even among targeted high-risk groups such as the elderly, pregnant women and HIV-infected adults.^34^  Vaccines for RSV are in advanced stages of development with phase 2 clinical trials underway in South Africa.^35^  For both influenza and RSV vaccination strategies targeting transmitters may be more cost-effective than risk-group based strategies.^36,37^ |
| Secondary objectives | 1. Objectives related to transmission dynamics, burden and health-seeking behaviour of influenza and RSV    1. To estimate the symptomatic fraction, the severity associated with symptomatic infections and the fraction of individuals with symptomatic infection seeking medical care among influenza- or RSV-positive cases by HIV serostatus and age    2. To estimate the SIR and length of shedding of influenza and RSV among HIV-infected and HIV-uninfected index cases and the rate of acquisition of influenza and RSV infection among HIV-infected and HIV-uninfected household members    3. To assess the role of asymptomatic infections in the household transmission of influenza an RSV    4. To estimate the correlation between individuals that seroconverted for influenza and RSV and tested positive at PCR for each of the two viruses | Data on the burden of mild and non-medically attended illness may be important for cost effectiveness of different vaccination strategies as mild illness is extremely common.^38^ These data are also important to understand the contribution of medically attended cases sampled through WHO recommended surveillance programmes as compared to total burden. Shedding duration and understanding the role of asymptomatic illness in transmission is relevant to guide control measures such as isolation or quarantine. The correlation between PCR and seroconversion is important to assist interpretation of serologic studies. |
|  | 1. Objectives related to respiratory virus characterization and evolution    1. Determine the contribution of specific influenza A and B subtypes or lineages to community burden of influenza    2. Determine the contribution of RSV-A and -B strains to the community burden of RSV    3. Determine the antigenic relatedness of influenza virus strains circulating within the community to the vaccine strains.    4. Determine the heterogeneity of influenza and RSV virus strains within household clusters and describe viral evolution within and between households as well as the association between virus strains and the duration of virus shedding and HIV status    5. Use molecular evolutionary analysis to better understand transmission networks associated with influenza virus and RSV spread within households and communities    6. Perform molecular analysis of the RSV F protein gene from circulating strains for naturally occurring polymorphisms associated with reduced susceptibility to therapeutic interventions    7. Assess the antigenic profile of RSV strains circulating within households and in the community    8. To identify the prevalence and duration colonization within the community by age group, vaccine status and over time, the transmission dynamics within a household, and the proportion of individuals that develop symptomatic infection for enterovirus, human metapneumovirus (hMPV), parainfluenza virus (PIV) 1-4, rhinovirus, coronaviruses (229E, OC43, NL63, HKU1), bocavirus, polyomavirus and adenovirus | Understanding the contribution of different serotypes and lineages to disease burden may inform targeted strategies such as vaccination. Understanding antigenic relatedness of virus strains and sero-response in the population may inform understanding of the interaction between these factors to drive virus evolution as well as the effect of this interaction on community outbreaks. Heterogeneity of virus strains within and between households can help understand the transmission bottleneck as well as virus evolution. Understanding transmission networks is useful to develop more accurate mathematical models and to guide potential interventions aimed at reducing transmission. Characterisation of RSV strains may inform choice of possible future vaccines and/or monoclonal antibodies. Understanding the transmission of respiratory viruses other than influenza and RSV can guide understanding of the burden and transmission of these viruses as well as virus-virus and virus-bacteria interactions. |
|  | 1. Objectives related to bacterial colonization and infection    1. To describe and compare nasopharyngeal pneumococcal loads in healthy individuals by age and over time, and how the loads may be altered by respiratory viral infection, HIV-status and vaccination-status    2. To determine the prevalence of pneumococcal DNA (*lytA)* in the blood of healthy individuals at a single point in time, and the correlation between detection in the blood and the presence of nasopharyngeal colonization and nasopharyngeal pneumococcal load    3. To identify the prevalence and duration of *B. pertussis* colonization within the community by age group, vaccine status and over time, the transmission dynamics within a household, and the proportion of individuals that develop symptomatic infection    4. To determine the prevalence of *N. meningitidis* colonization within the community by age group at a single point in time each year and the predominant serogroups being carried within a household    5. To determine the prevalence of *C. diphtheriae* colonization within the community by age group at a single point in time each year and the characteristics of diphtheria strains being carried within a household    6. To determine the prevalence of *S. pyogenes* colonization within the community by age group at a single point in time each year and the characteristics of *S. pyogenes* strains being carried within a household    7. To describe the composition of the nasopharyngeal microbiota in healthy individuals, by age and over time, and how this changes with the development of symptomatic and asymptomatic infection in the post-pneumococcal conjugate vaccine (PCV) era | The pneumococcal conjugate vaccine (PCV) was introduced into the South African immunization programme in 2009, associated with substantial reductions in serotype-specific pneumococcal disease and increases in serotypes not included in PCV.^39^ Reduced dose schedules, relying on indirect effects are being considered for pneumococcal vaccination in South Africa. Data on burden and household transmission in the post-vaccination era will be key to guiding these decisions.  In 2009, South Africa replaced the whole-cell pertussis vaccine with the acellular vaccine, likely a contributing factor to recent increased pertussis incidence.^40^ Understanding community burden and transmission may inform recommendations to control outbreaks.  Invasive disease (meningitis and bacteraemia) caused by the fulminant bacterial pathogen *Neisseria meningitidis* (meningococcus) remains a significant problem worldwide. The ecological niche for *N. meningitidis* is the human nasopharynx/throat and carriage rates are approximately 5-10% during endemic disease but may be significantly higher during epidemics or in closed communities^41^. Carriage data are thus important in trying to further our understanding of disease and potentially improve patient management.^41^  *C. diphtheriae* can cause life-threatening infection of the throat as well as cardiac and neurologic complications. The most common clinical presentation of *C. diphtheriae* infection is an upper respiratory tract infection, most commonly involving the tonsils, pharynx, larynx, or nasal mucosa^42^. The incubation period is usually between 2 to 5 days, and humans are the only known reservoir.^42^  *S. pyogenes* causes a range of infections including pharyngitis and may be asymptomatically carried in the throat.^43^ Infection may lead to acute rheumatic fever and rheumatic heart disease which is a notifiable medical condition in South Africa.  At any one point in time, the nasopharynx of an individual is inhabited by a complex microbiome of pathogens. Use of polymerase chain reaction (PCR) to determine the aetiology of respiratory tract infections^44,45,46^ has highlighted the prevalence of polymicrobial respiratory tract infections^47–49^. The interaction of these co-existing pathogens in the upper respiratory tract plays an important role in the bacterial loads of the organism in the nasopharynx, and development of disease. |
|  | 1. Objectives related to important co-infections such as tuberculosis or HIV    1. To measure the annual incidence of tuberculosis infection in individuals living in study households and assess risk factors (including incident and prevalent HIV) for acquiring tuberculosis infection.    2. To evaluate whether infection with respiratory viruses leads to increased incidence of tuberculosis infection or disease    3. To evaluate whether tuberculosis disease is associated with increased influenza transmission in households | The morbidity and mortality  associated with a diagnosis of tuberculosis is severe, in South Africa approximately 24% of all patients with TB die.^50^ Despite this burden, details on where transmission and acquisition of tuberculosis occur are scanty.  As tuberculosis infection precedes symptomatic disease, data on prevalence and incidence of tuberculosis infection are scarce; there are few contemporaneous studies of individuals from Southern Africa reporting rates of tuberculosis infection in different settings.  Moreover, an occasional prospective study reports incident tuberculosis infection, as part of a clinical trial in a well selected sub-group. |
|  | 1. Objectives related to housing quality and exposure to indoor air pollution    1. To document housing quality in all dwellings    2. To determine the main fuels used for cooking and space and water heating;    3. To measure levels of indoor air pollution (particulate matter and carbon dioxide) in the study dwellings;    4. To measure levels of dust deposition indoors;    5. To examine dampness and mould in dwellings as a risk factor for respiratory symptoms    6. To ascertain the impact of housing quality and exposure to indoor air pollution on tuberculosis infection and on household transmission of respiratory viruses and bacteria | Despite the rollout of a massive electrification programme over the past two decades, large numbers of South African households continue to make use of polluting fuels for cooking and space heating.^29^ Evidence reviews have shown that the risk of pneumonia in young children is increased by exposure to unprocessed solid fuels by a factor of 1.8.^51,52^ In the South African context therefore, an examination of the role of exposure to domestic air pollution in respiratory disease transmission is of high importance. |

Supplementary table 2: Comparison of characteristics of individuals who participated in the study compared to those who did not participate within included households, a rural and an urban site, South Africa, 2016-2018

| **Characteristic** | **Participated**  **n (%)**  **N=1684** | **Did not participate**  **n (%)**  **N=511** | **p** |
| --- | --- | --- | --- |
| Rural site (vs urban) | 849 (50) | 310 (61) | <0.001 |
| Year  2016  2017  2018 | 542 (32)  577 (34)  565 (34) | 144 (28)  197 (39)  170 (33) | 0.131 |
| Age group (years)  <1  1-4  5-14  15-44  45-64  ≥65 | 37 (2)  245 (15)  546 (32)  588 (35)  195 (12)  73 (4) | 9 (2)  55 (11)  115 (23)  250 (49)  54 (11)  28 (5) | <0.001 |
| Female sex | 1009 (60) | 257 (50) | <0.001 |

Supplementary table 3: Comparison of individual and household-level characteristics of individuals lost to follow-up for twice-weekly visits to those with complete follow-up, a rural and an urban site, South Africa, 2016-2018

| **Characteristic** | **Complete follow-up** | **Lost to follow-up** | **p** |
| --- | --- | --- | --- |
|  | **n (%)** | **n (%)** |  |
| **Household level characteristics** | **N=321** | **N=6** |  |
| Year  2016  2017  2018 | 99 (31)  107 (33)  115 (36) | 1 (17)  2 (33)  3 (50) | 0.699 |
| Number of household members  3-5  6-10  >10 | 190 (59)  119 (37)  12 (4) | 6 (100)  0  0 | 0.130 |
| Number of rooms  1-4  5-9  ≥10 | 135 (42)  173 (54)  13 (4) | 2 (33)  3 (50)  1 (17) | 0.315 |
| Number of rooms for sleeping  1-2  3-4  ≥5 | 170 (53)  140 (44)  11 (3) | 3 (50)  3 (50)  0 | 0.874 |
| Crowding (>2 people/room) | 158 (49) | 2 (33) | 0.441 |
| Child<5 years in house | 224 (70) | 1 (17) | 0.005 |
| Smoke in house | 69 (21) | 2 (33) | 0.486 |
| Main water source tap inside (vs tap outside) | 152 (47) | 2 (33) | 0.495 |
| Handwashing place with water in house | 258 (80) | 6 (100) | 0.227 |
| Main fuel for cooking  Electricity  Wood  Paraffin (kerosene)/gas/other | 237 (75)  70 (22)  10 (3) | 4 (80)  1 (20)  0 | 0.911 |
| **Individual level characteristics** | **N=1,605** | **N=79** |  |
| Age group (years)  <1  1-4  5-14  15-44  45-64  65+ | 33 (1)  234 (15)  534 (33)  546 (34)  190 (12)  68 (4) | 4 (5)  11 (14)  12 (15)  42 (53)  5 (6)  5 (6) | 0.001 |
| Female sex | 960 (60) | 49 (62) | 0.695 |
| Year  2016  2017  2018 | 521 (32)  546 (34)  538 (34) | 21 (27)  31 (39)  27 (34) | 0.492 |
| HIV status^a^  Uninfected  Infected  Unknown | 1,324 (85)  235 (15)  46 | 55 (80)  14 (20)  10 | 0.239 |

^a^% and p value among individuals with known HIV status (p value including unknown HIV status<0.001)

Supplementary Figure 1: Timing of study procedures in relation to enrolment, follow up and season, a rural and an urban site, South Africa, 2016-2018 a)rural site b) urban site

A

B
